# Asthma: Is It Time for Monocytes to Share the Spotlight?

**DOI:** 10.3390/cells15141233

**Published:** 2026-07-08

**Authors:** Harissios Vliagoftis, Nami Shrestha Palikhe

**Affiliations:** Department of Medicine and Alberta Respiratory Centre, University of Alberta, Edmonton, AB T6G 2S2, Canada

**Keywords:** asthma, monocytes, airway

## Abstract

The evolving understanding of the regulation of allergic airway inflammation has offered new approaches for asthma therapy. For example, our understanding of the role of alarmins, Th2 cytokines and eosinophils has allowed the development of biologics that have revolutionized therapy for severe asthma. However, many questions remain and several of our patients are still not controlled with the current available therapies. Many immune cells have been implicated in asthma pathophysiology, but one cell that is missing from these studies is the monocyte. Monocytes (Mos) are bone marrow-derived cells that circulate in the blood and develop into macrophages and/or dendritic cells following migration to peripheral tissues. Macrophages (Møs) and dendritic cells have been implicated in the development and progression, of allergic airway inflammation, but also in tissue repair after inflammation. Recent studies in animal models suggest a major role for Mos in allergic airway inflammation, primarily through their ability to mediate recruitment of eosinophils and/or neutrophils to the airways. However, there is little information regarding the role of monocytes in human asthma. Here we review the literature regarding the presence and functions of peripheral blood and airway Mos in human asthma and suggest further work that needs to be done to consolidate the information on Mo functions. Studies show changes in Mo numbers and activation status in the peripheral blood of patients with asthma, changes that in many cases correlate with disease severity and/or activity. Studies also show altered phenotype of Mos present in the airways of patients with asthma. Detailed human studies need to be performed, if possible, studies that include therapeutic interventions, to allow for a full understanding of the role of Mos in asthma.

## 1. Introduction

The evolving understanding of the regulation of allergic airway inflammation over the past 30 years has fostered the development of effective newer asthma treatments, such as monoclonal antibodies targeting inflammatory mediators. These new therapeutic modalities have made a real impact in the treatment of severe asthma. Many immune cells, such as eosinophils, Th2 lymphocytes, T regulatory cells, type 2 innate lymphoid cells (ILC2s), dendritic cells, and mast cells, in addition to parenchymal cells, have been implicated in asthma pathophysiology. However, we still do not have the complete picture of asthma pathophysiology, especially as related to human disease. Improved understanding would benefit a significant number of subjects with asthma that are still not controlled with current therapies.

Our understanding of the role of specific cell types or immune cell phenotypes in allergic airway inflammation comes primarily from murine studies [1]. Little of this knowledge can be directly proven in human *in vivo* models, but in vitro or ex vivo studies that support murine study results offer validation that the same mechanisms are also functional in human disease.

Monocytes (Mos) circulate in blood and migrate into tissues, especially inflamed tissues, where they may differentiate into macrophages (Møs) and/or dendritic cells (DCs) [2]. Møs and DCs have been extensively studied in asthma [3], but the role of Mos is relatively unexplored. Recent animal studies have suggested that Mos may play a crucial, and hitherto overlooked, role in the pathogenesis of Th2 and possibly also Th1 disease in the airways [4,5,6].

## 2. Role of Mos in Asthma—Lessons from Murine Studies

Recent animal studies have suggested a major role for Mos in allergic airway diseases. Depletion of blood Mos in mice reduces eosinophilic airway inflammation and airway hyper-responsiveness (AHR) in models of asthma [4,5]. In addition, Mos facilitate neutrophil recruitment to the lung [6], an effect that may play a role in neutrophilic asthma. Unfortunately, these results have not been followed with more detailed studies that would fully delineate the mechanisms of these effects. Nevertheless, additional supporting evidence continues to highlight the importance of Mos in obstructive airway diseases.

Mo recruitment to murine airways by diesel exhaust particles (DEPs) in a CCR2-dependent manner may mediate subsequent eosinophil recruitment [7]. Specifically, DEPs are taken up by alveolar Møs and release Mo chemotactic factors that mobilize Mos from the bone marrow (BM) [8] and facilitate their recruitment to the airways in models of allergic airway inflammation [7,8]. In mice, Mo recruitment to the airways is also required for AHR development in response to influenza infections [9], an observation that suggests that Mos may also be important contributors to virally induced asthma exacerbations. In addition, nasal RSV infection in house dust mite (HDM)-sensitized mice increases AHR, an event that is again dependent on Mo recruitment to the airways [10]. Finally, blockade of the CCL2/CCR2 pathway decreases inflammatory Mo recruitment to the peritoneal cavity following sensitization with OVA intraperitoneally and subsequently decreases allergic sensitization and eosinophil accumulation in the airways [11].

These results indicate that Mos recruited to the airways may mediate recruitment of other inflammatory cells, such as eosinophils and/or neutrophils, as well as mediate, directly or indirectly, AHR even though the mechanisms for these effects are not very clear and the exact contribution of Mos themselves versus Møs or DCs derived from these Mos is also not fully understood.

Despite these promising findings, the role of Mos in asthma remains largely unexplored in humans, leaving a significant gap in our understanding of the disease and limiting our therapeutic options for patients not controlled with current therapies.

## 3. Mos, Mo Subsets, and Their Functions in Mice and Humans

Almost 60 years ago it was shown that peripheral blood Mos originate from bone marrow progenitors and circulate through the blood with a short half-life before migrating into peripheral tissues giving rise to tissue Møs [12], while later it was described that Mos also give rise to DCs in the blood and tissues. Circulating human Mos are a heterogeneous population [13,14]. They are classified by flow cytometry as CD14^++^CD16^−^ (classical; CMo), CD14^++^CD16^+^ (intermediate; IMMo), and CD14^+^CD16^+^ (non-classical; NCMo) Mos [15,16]. Recent human *in vivo* studies have shown that Mos egress from BM as CMos and subsequently differentiate into IMMos and NCMos [17]. Mos can migrate into peripheral tissues under non-inflammatory conditions and in that case, they retain most of their characteristics, but upregulate MHCII, and serve to survey the tissue and transport antigens to draining lymph nodes [18]. Mos recruited into peripheral tissues during inflammation are highly plastic cells that are programmed by the tissue microenvironment [19,20]. In subsequent chapters we will present the available data on Mo changes and plasticity when recruited into the lungs in allergic airway diseases.

Mo subsets exhibit different properties regarding their immune and other functions, properties that depend among others on differential methylation of immune genes [21]. CMos are highly migratory, express elevated levels of chemokine receptors, and are more effective in production reactive oxygen species and pro-inflammatory mediators compared to other subsets [22]. IMMos are better equipped for antigen presentation and respond well to TLR activation. NCMos are considered anti-inflammatory; they patrol the endothelium and are important for maintenance of vascular homeostasis and for recognition and clearance of pathogens [23].

Mouse peripheral blood Mos are divided into two subsets referred to as classical Mos (Ly-6C^high^CCR2^+^CX3CR1^lo^ cells) and non-classical Mos (Ly-6C^lo^CCR2^−^CX3CR1^high^ cells). Even though there are significant differences between human and mouse Mos, there are data suggesting an equivalence between phenotypes [24]; human CMos that comprise approximately 95% of human peripheral blood Mos correspond to Ly-6C^high^ murine Mos that are approximately 50% in peripheral blood, while NCMos correspond to Ly-6C^lo^ murine cells.

## 4. Mos in Healthy Human Airways

Mos migrate to the lungs and in addition to becoming Møs and replenishing lung Mø niches, they can also retain their Mo characteristics. An interesting study to characterize lung and airway Mos in normal individuals showed that airway samples were enriched in IMMos compared to blood and that peripheral airways had increased IMMo numbers compared to more central airways [25]. In addition, airway IMMos showed a phenotype resembling DCs (CD1c expression) but not Møs (no changes in CCR2 or CD163 expression), similar to peripheral blood IMMos which are known to be more efficient antigen-presenting cells than other Mo subsets [26,27]. Furthermore, airway Mos, including IMMos, produced less cytokines compared to blood Mos after LPS-mediated activation [25]. There is also evidence that in healthy individuals, blood and BAL CMos increase from childhood to adulthood and then decrease in older individuals [28]. These data suggest that airway Mos may have primarily an immunoregulatory instead of an inflammatory role in healthy lungs. This role may be different for Mos present in inflamed lungs.

A multicolor flow cytometry approach allowed the identification of Mos in the airways and lung tissue and their separation from Møs and DCs [26]. All three subsets of Mos were identified with increased numbers of IMMos compared to blood and very few NCMos, something seen in both the airways and lung tissue. They were also able to show some changes in chronic smokers and subjects with idiopathic pulmonary fibrosis (IPF).

Several studies have developed methods to identify different Mo populations in human lung tissue, BAL, and lymph nodes and differentiate them from DCs, alveolar macrophages (AMs) and other monocytic cell populations [29,30]. In another study researchers analyzed the phenotype of mononuclear cells from 72 donated lungs that were not used for transplantation [30]. They selected lungs from donors without a history of chronic lung disease, reasonable lung function, no evidence for lung infiltrates, no long history of smoking and limited time on a ventilator before lung removal. Having access to intact lungs they were also able to separate cells found in the intravascular space versus cells that were into the lung parenchyma. Mo populations within the lung parenchyma and airways had several different characteristics than cells in the vascular compartment with a significant difference in CD206 expression that was not detected on Mos present in the vasculature. Extravascular CD14^+^ Mos had similar morphology and size with intravascular cells and similar CD16 and CCR2 expression, but they had higher levels of CD11c, HLA-DR and CCR7.

## 5. Mos, Mo Subsets, and Asthma in Humans (Current Knowledge and Knowledge Gaps)

**Figure 1:**
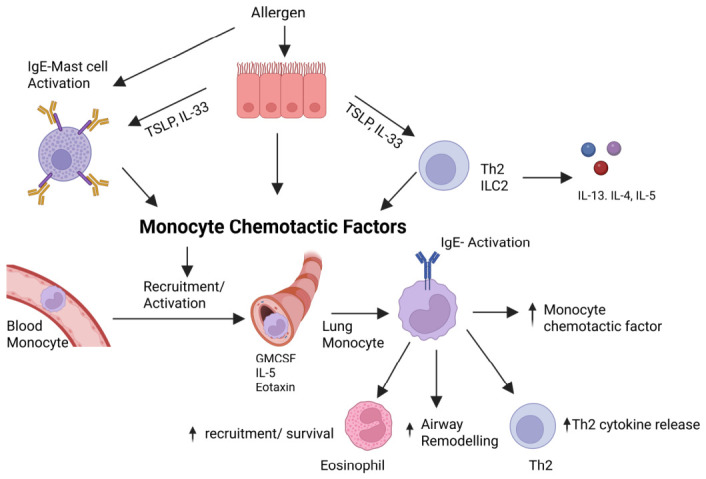
Mechanisms inducing Mo accumulation in the lungs/airways and potential biological consequences of recruited Mo in allergic airway inflammation. IgE-mediated mast cell activation and allergen-induced epithelial cytokines (TSLP, IL-33, and IL-25) promote the release of Mo chemotactic factors through Th2-mediated pathways, leading to the recruitment and activation of blood and lung Mos. Activated lung Mos enhance eosinophilic inflammation through the release of GM-CSF, IL-5, and eotaxins, promoting the recruitment and survival of eosinophils and Th2 cells. In addition, crosslinking of the high affinity IgE receptor on Mo themselves releases Mo chemotactic factors, contributing to ongoing airway inflammation.

The association of fundamental allergy pathways with Mo chemotactic or other factors that affect Mo growth, survival and activation suggests a major Mo involvement in asthma. IgE-mediated mast cell activation leads to Mo chemotactic factor release [31,32]; furthermore, crosslinking of the high affinity IgE receptor on Mos themselves releases inflammatory factors, including Mo chemotactic factors [33], and prolongs Mo survival [34]. IL-4 and IL-13, important Th2 cytokines involved in asthma pathophysiology, mediate Mo chemotactic factor release from the airway epithelium [35,36], but can also directly prolong Mo survival and induce Mo activation [37,38]. TSLP, an alarmin important in allergic inflammation, induces the release of Mo chemotactic factors from stroma cells [39,40], lung fibroblasts [41] and mast cells [42] and can directly activate Mo inflammatory responses [43]. In addition, Mos may produce eosinophil survival factors GM-CSF [44] and IL-5 [45,46], increase the capacity of T cells from atopic subjects to produce IL-5 [47], and release eotaxins [48,49]; they may therefore promote eosinophil recruitment and survival in the airways. A summary of the mechanisms that may induce Mo accumulation in the lungs/airways and the potential biological consequences of recruited Mo in allergic airway inflammation is shown in Figure 1.

However, until recently direct evidence for Mo involvement in asthma was limited. Old reviews suggest that airway Møs derive from blood Mos and present no direct evidence of Mo presence in the airways [50,51]. Reports of airway mucosa infiltration with Møs resembling peripheral blood Mos, very likely represent tissue Mos [52], which may have inflammatory and/or immunoregulatory roles [18].

### 5.1. Blood Mos in Patients with Allergic Diseases

Several studies report alterations of numbers and or phenotype/activation status of Mos and Mo subsets in peripheral blood in patients with asthma. The parameters studied vary between studies, but the literature suggests associations between allergic airway diseases and peripheral blood Mos. Unfortunately, none of these studies attempt to address the issue of causality, which is always difficult to address in human non-intervention studies.

There is evidence for increased numbers of peripheral blood Mos (CD14^+^ cells) in asthma versus healthy controls [53], although changes in Mo numbers in asthma are not a consistent finding [54]. In subjects with mild asthma baseline characteristics of peripheral blood Mo populations correlate with development of single or dual responses after an allergen challenge [55], while these characteristics change 24 h post-allergen challenge in some patients, indicating that inhaled allergen directly affects Mos in the periphery. Total blood Mos were increased only in patients with severe asthma compared to healthy controls, while CD16 expression was decreased in IMMos and NCMos in subjects with severe asthma [56]; no big changes were seen in other Mo populations. In NCMos, CCR2 was decreased and CX3CR1 increased in all subjects with asthma compared to healthy controls [56].

Numbers of Mo subsets also vary depending on asthma severity. Patients with severe asthma had higher numbers of blood CMos than non-severe asthma [57,58] and also higher levels of serum CCL2 [57]. CMos from subjects with severe asthma generated higher levels of extracellular traps (ETs) in response to IFNγ and LPS compared to cells from patients with non-severe asthma and these ETs in turn were able to induce neutrophil chemotaxis, promote neutrophil cytokine release and activate ILC1 and ILC3, suggesting a role for CMo ETs in asthma pathophysiology [57].

Peripheral blood IMMos also increased with increased asthma severity [59], while treatment of asthma exacerbations decreased IMMo numbers. The percentage of IMMos in peripheral blood was also higher in subjects with asthma and HDM sensitivity responding to an inhalation allergen challenge with HDM extracts compared to non-responders and healthy controls [60]; in this case IMMo numbers also correlated with AHR. IMMos also had the highest expression of CCR4, a receptor for CCL17, among Mo subsets [60] and their numbers decreased 24 h after allergen challenge in responders, a change that correlated inversely with CCL17 concentration. We have recently shown that CCL17 is one of the Mo chemotactic chemokines that increase in the airways after an inhalation allergen challenge and CCR4 is a major receptor mediating peripheral blood Mo chemotaxis in response to induced sputum supernatants from subjects with mild asthma [61]; in the same study we showed that CCR5, a receptor activated by CCL4 that also increases post-allergen challenge, also mediates Mo chemotaxis to induced sputum supernatants. Both CCR4 and CCR5 have been implicated in asthma pathophysiology [62,63] and may mediate Mo recruitment to the airways.

Patients with severe asthma show evidence of increased numbers of activated IMMos in the peripheral blood, as shown by increased surface expression of Protease-Activated Receptor-2 (PAR-2) [54]; similar increased PAR-2 expression on IMMos is seen in patients during an asthma exacerbation and in patients with mild asthma following an inhalation allergen challenge [64]. Increased airway inflammation may be the cause of this increased PAR-2 expression, since treatment of the asthma exacerbation allows activated peripheral blood Mos to return to the levels of patients with stable asthma [64]. Another group has also shown increased PAR-2 expression on Mos from patients with allergic airway diseases [65] and they also showed that PAR-2-mediated Mo activation leads to the release of pro-inflammatory factors. It is also interesting that the total peripheral blood Mos were decreased during exacerbations [64], suggesting that these activated Mos may be recruited to inflamed tissues, primarily lungs/airways, during an exacerbation, possibly through similar mechanisms that may recruit Mos to the lungs after a successful inhalational allergen challenge [60]. Increased Mo recruitment to the airways may have pro-inflammatory effects, especially if these cells are IMMos which have higher expression of antigen processing and presentation genes, are able to induce increased T cell proliferation in co-culture assays, and have increased expression of genes involved in oxidative stress [26], compared to other Mo subsets.

Other studies have also shown increased Mo activation in the peripheral blood of subjects with allergic airway diseases. In children, activated Mo increase in blood during asthma exacerbations [66]. Peripheral blood CD14^+^ Mos from subjects with symptomatic allergic rhinitis had higher expression of CD11c compared to cells from asymptomatic sensitized subjects and healthy controls [67]; on the other hand FcεRI expression was higher in all atopic individuals compared to healthy controls, but showed no difference between subjects with symptomatic disease and asymptomatic sensitized individuals. Peripheral blood Mos from patients with allergic airway diseases express higher levels of substance P and neurokinin-1 receptors [68], suggesting a potential role of Mos in neuroinflammation in patients with allergic diseases. Mos from these patients have also been shown to have higher expression of CD1d compared to cells from healthy controls [69], suggesting increased capacity for communication with invariant NKT cells and possibly increased pro-inflammatory effects.

### 5.2. Mos in the Airways of Patients with Asthma

Mo chemotactic factors are increased in the airways in patients with asthma. CCL4 and CCL17 increase in induced sputum after an inhalation allergen challenge and support Mo chemotaxis through at least CCR2/CCR5 and/or CCR4 [61]. Another Mo chemotactic factor, CCL13, is increased in biopsies and BAL from asthmatics versus normal controls [70], is increased in epithelium and immune cells in patients with asthma [71], and is found in high levels in induced sputum [72]. These chemotactic factors may be responsible for the increased numbers of Mod seen in the airways in a number of studies.

Induced sputum from patients with asthma has increased numbers of CMos compared to sputum from healthy controls [58]; CMo numbers were also increased in neutrophilic versus paucigranulocytic asthma, while CD206^-^ Mo proportion and numbers were increased in neutrophilic versus eosinophilic asthma. Mos in the sputum of patients with asthma have increased inflammatory markers and their numbers correlate with other indicators of inflammation [73].

Mos accumulate in the nasal mucosa of atopic individuals following a nasal allergen challenge [74,75]. In children, activated Mos are present in high numbers in the lower airways in fatal asthma [75], where Mo cellular aggregates appear to obstruct bronchioles. Mos accumulating in the upper airways following an allergen challenge [74] or in the lower airways during fatal asthma [75] express S100A8/A9, and immunohistochemical staining for this marker differentiates them from alveolar macrophages. S100A8/A9 is an alarmin modulating the inflammatory response in many organs [76] and has been associated with asthma pathogenesis [77]. Other studies showed increased numbers of CD14^+^ cells in the BAL in symptomatic asthma [78], and in bronchial washes from patients undergoing an allergen challenge [79]. When Mos and Møs were evaluated together there was no difference in numbers between subjects with fatal asthma and those with mild/moderate disease [80].

Having information about recruitment and even activation status of Mos in the airways does not answer the question whether their presence or activation status are important for asthma pathophysiology, and whether they are responsible for the airway inflammatory changes seen on asthma, or if their presence might just be an epiphenomenon of allergic airway inflammation. In human studies the role of Mos often cannot be shown directly but needs to be inferred from the presence and activation characteristics of Mos in subjects with asthma of various phenotypes and severity. Recent technological advances, such as single cell RNA sequencing (scRNAseq), provide new ways to evaluate Mos and obtain clues about their functions in asthma.

One such study recruited allergic asthmatics (AAs) and allergic controls (ACs; allergic individuals that do not have asthma) and performed segmental allergen challenges; these subjects had BAL and bronchial brushing obtained before and after an allergen and a placebo challenge [81]. In both groups the number of mononuclear phagocytes (MNPs) increased after challenge. Sub-clustering of these MNPs by scRNAseq identified 14 clusters of DCs, macrophages and monocyte-derived cells (MCs) based on published transcriptional characteristics of these cells. Five clusters were annotated as MCs based on high expression of canonical monocyte genes (i.e., CD14, VCAN, and FCN1), consistent with the well-described plasticity of MCs [19,20]. It is interesting that different clusters of Mos accumulated in the airways of AA versus AC individuals. In addition to accumulation of different clusters/phenotypes of Mos, specific clusters showed phenotypic differences that suggest that Mos play different roles in the airways of asthmatic and non-asthmatic individuals. In ACs, the MC phenotype was characterized by autocrine production of factors important in endocytic clearance, Mø differentiation and survival, and expression of trophic factors promoting angiogenesis and tissue repair. In contrast, in asthmatic individuals, IL-4/IL-13 signaling via STAT6 induced a pathogenic MC phenotype characterized by upregulation of genes involved in inflammatory signaling, antigen presentation, and pathologic airway remodeling. It is also interesting that Mos within a specific cluster responded differently to allergen challenge in AA versus AC individuals; for example, cells from cluster MC2 from AA individuals upregulated genes involved in recruitment of T2 cells, while MC2 cells in AC subjects upregulated genes associated with Mo survival and phagocytic function. Such observations suggest that it is not just whether Mos or Mo subsets are present in the airways, but it is the specific inflammatory or anti-inflammatory pathways activated in these cells that may define their role in various conditions.

Another scRNAseq study of mucosal biopsies from patients with asthma [81] also showed increased Mo presence after segmental allergen challenge; CCR2-expressing Mos were prominent among accumulating Mos, indicating that CCR2 ligands may be important for Mo recruitment into the lungs/airways. Furthermore, Mos in the asthmatic airways after allergen challenge upregulated genes involved in pathologic remodeling, suggesting a detrimental role for these cells in the airways. This observation is supported by the fact that Mos in the peripheral blood of patients with severe asthma exhibit a pro-inflammatory phenotype [82].

Studies that analyze patients experiencing natural asthma exacerbations have an advantage over those using inhaled allergen extracts, as the mechanisms driving increased inflammation during asthma exacerbation are more physiological than those induced by allergen challenges. One such study used scRNAseq to show increased numbers of Mos in the BAL of subjects with asthma exacerbations as well as the presence of CCL5, a Mo chemotactic factor [83]. The study identified many clusters of Mos, in addition to Mø clusters. One of the Mo clusters was among the most activated cells during the asthma exacerbations with activation of many pathways including mobility, inflammatory response and cytokine production pathways as well as the CXCR4 signaling pathway, another receptor for Mo chemotactic factors. Mo clusters also showed upregulation of factors associated with Th1 responses during natural asthma exacerbations (i.e., IL-27 and T-bet) suggesting that Mos may contribute to viral pathways that are often activated during asthma exacerbations.

A limitation of many of the omics studies is the difficulty of generating longitudinal data or analyzing large cohorts of patients that would allow us to compare between different disease phenotypes or disease severity. Studies that have attempted to analyze larger cohorts exist, and have generated very interesting data, for example, on various mechanisms of asthma exacerbations [84]. Similar studies using scRNAseq might be able to generate more detailed data on the involvement of specific cell types in these asthma phenotypes and possibly other phenotypes that have not been elucidated yet.

### 5.3. Studies on the Effects of Biologics on Mo Presence and Activation Status in the Airways

Today we have some excellent models to study immunology in vivo in humans; these are patients with specific diseases treated with biologics against important immune molecules/pathways. A number of these biologics are used in asthma today; these biologics target IgE-activated pathways, as well as IL-5-, IL-4/IL-13- and TSLP-activated pathways. Studies on the effects of these biologics on the numbers and activation status of Mos in the peripheral blood and the airways could improve our understanding of the roles of Mos in allergic airway diseases.

PBMCs from eight patients with severe asthma treated with biologics were analyzed by scRNAseq before, as well as 1 and 6 months after, initiation of therapy [85]. Multiple myeloid cell subgroups could be identified including CMos, IMMos, NCMos, unclassified Mos, conventional DCs and plasmacytoid DCs. Treatment with mepolizumab, reslizumab or dupilumab altered the composition of CMos in blood from cells expressing high levels of IL1b to cells expressing S100A proteins but did not affect the general categories of the other myeloid cells. Furthermore, all these biologics decreased expression of molecules in the NfkB pathways across all cell types. The significance of S100A increase in Mos after therapy is not clear. S100A proteins are associated with neutrophil activation and whether these changes mean that T2 skewing of the immune environment is decreased following therapy with biologics, or if there are other explanations for these changes, is not clear from current studies. This change could also be the result of specific Mo subsets moving into or out of the affected organs due to changes in Mo trafficking following therapy.

A study, using scRNAseq on peripheral blood cells of patients with asthma treated with omalizumab or mepolizumab, and available as a preprint (medRxiv preprint; https://doi.org/10.1101/2025.04.16.25325934, assessed on 7 July 2026), did not find significant differences in Mos among subjects that responded or did not respond to therapy, but identified markers in pDC that could predict responsiveness. Full publication of this study might better define these changes.

IgE, IL-4/IL-13 and TSLP are dominant pathways in asthma pathophysiology and biologic agents interrupting these pathways are very successful severe asthma therapeutics [86]; their effectiveness may also be associated with their ability to prevent Mo activation and Mo chemotactic factor production. Overall, these studies support the concept that in asthma, Mos may release, or activate other cells to release, pro-inflammatory mediators, facilitating recruitment and differentiation of eosinophils and neutrophils, while they may also facilitate asthma development and symptom manifestation through differentiation into DCs and/or Møs. However, direct evidence of Mos having an important role in human asthma is lacking. The information discussed above is presented in a diagrammatic form in Table 1.

## 6. Conclusions

Understanding the role of Mos in human disease is a complex process. Although it may be more semantic than clinically important, it is very difficult to separate the effects that Mos elicit on their own, versus effects elicited by their progeny, such as Møs and DCs, that can develop from Mos recruited to the airways.

As was also the case with eosinophils, or TSLP, more information will come from human studies using biologics or small molecules that affect Mo development, recruitment and/or activation. Blocking a number of different chemokine receptors that can recruit Mos, such as CCR4 [63,87,88], CCR2 [7,11], or CXCR4 [89,90] in mice or CCR2 [91] in cynomolgus monkeys, decreases allergic airway inflammation in animal models. However, no such studies have been done so far in humans. There are a number of chemokine/chemokine receptor-blocking biologics and small molecules that are already in use, or are coming soon, for other human conditions. For example, therapeutics blocking CCR4 and CCR5 are in use [92,93,94,95] and show promise in inflammatory conditions [96,97]. Some of these biologics have good safety profiles and it may be worth testing them on asthma. It would also be interesting to analyze data from studies of these molecules in other conditions for possible effects on airway inflammation that was present in participating subjects.

In conclusion, the current literature is focusing on omics approaches to better understand the role of Mos in human airway inflammation, while direct approaches to visualize and study Mos are limited and have not been as informative. Although omics approaches are very powerful, they require validation using other methods to directly observe the different cell types, quantify them, validate their activation status and use them ex vivo for mechanistic studies. Such studies will facilitate subsequent in vivo human studies to test the role of Mos using interventions that prevent their recruitment and/or activation.

## Figures and Tables

**Table 1:** Studies on the role of peripheral blood and lung/airway Mos in allergic airway inflammation.

Finding	Model	Species	Reference
Blood Mo depletion reduces eosinophilic airway inflammation and AHR	Allergic airway inflammation	Mice	[4,5]
DEP-induced Mo recruitment may drive eosinophil recruitment	DEP-induced airway inflammation	Mice	[7]
CCL2/CCR2 blockade reduces inflammatory Mo recruitment, allergic sensitization, and airway eosinophilia	OVA allergy model	Mice	[11]
Airway Mo recruitment is required for influenza-induced AHR	Influenza infection	Mice	[9]
RSV infection in HDM-sensitized mice increases AHR via Mo recruitment	Asthma exacerbation	Mice	[10]
Mos promote neutrophil recruitment to the lungs	CCL2-CCR2-mediated lung inflammation	Mice	[6]
RSV infection in HDM-sensitized mice increases AHR via Mo recruitment	Asthma exacerbation	Mice	[10]
CCL2/CCR2 blockade reduces inflammatory Mo recruitment, allergic sensitization, and airway eosinophilia	OVA allergy model	Mice	[11]
Blocking monocyte-recruiting chemokine receptors (CCR4, CCR2, or CXCR4) reduces airway inflammation in animal models	Airway inflammation	Mice	[63,87,88,89,90,91]
Peripheral blood Mos are increased in asthma	Asthma	Human	[53]
Intermediate Mos in blood increase with asthma severity	Asthma	Human	[54]
Increased CMos and serum CCL2 in severe asthma compared to non-severe asthma	Severe asthma	Human	[57]
Baseline peripheral blood Mo characteristics correlate with single or dual responses following allergen challenge, suggesting a direct effect of inhaled allergens on Mo populations	Allergen challenge	Human	[55]
Altered peripheral blood CMo phenotype after treatment with mepolizumab, reslizumab or dupilumab in severe asthma—transition from CMo expressing high levels of IL1β to cells expressing S100A proteins	Asthma	Human	[85]
Increased CMos in induced sputum of asthma patients and neutrophil asthma	Asthma sputum	Human	[58]
Mo and CCL5 increase in the BAL during asthma exacerbations—evidence for involvement of the CXCR4 signaling pathway	Asthma exacerbation	Human	[83]
Inhalational allergen challenge in patients with mild asthma increases Mo chemotactic factors in induced sputum (primarily CCL4 and CCL17)	Allergen challenge	Human	[61]
Increased CMos in induced sputum of asthma patients and neutrophil asthma	Asthma sputum	Human	[58]
Mos accumulate in the nasal mucosa after nasal allergen challenge	Allergen challenge	Human	[74,75]
Increased airway Mo-derived cells after allergen challenge	Allergen challenge	Human	[81]
Recent technological advances such as scRNAseq provide powerful new approaches to evaluate Mos and better understand their potential roles in allergic disease	Asthma	Human	[81,82,83,84]
Reviews the evidence of biologics for Mo activation and recruitment of other inflammatory cells and chemotactic factors	Asthma	Human	[86]

## Data Availability

Data generated through literature review are available directly from the authors.
